# *miR-92a* Suppresses Mushroom Body-Dependent Memory Consolidation in *Drosophila*

**DOI:** 10.1523/ENEURO.0224-20.2020

**Published:** 2020-08-14

**Authors:** Tugba Guven-Ozkan, Germain U. Busto, Jae-Yoon Jung, Ilaria Drago, Ronald L. Davis

**Affiliations:** 1 Department of Neuroscience, The Scripps Research Institute Florida, Jupiter, FL 33458; 2 Department of Pediatrics/Systems Medicine, Stanford University, Stanford, CA 94305

**Keywords:** Memory consolidation, memory suppressor gene, microRNA, kinesin heavy chain

## Abstract

MicroRNAs (miRNAs) fine tune gene expression to regulate many aspects of nervous system physiology. Here, we show that *miR-92a* suppresses memory consolidation that occurs in the αβ and γ mushroom body neurons (MBns) of *Drosophila*, making *miR-92a* a memory suppressor miRNA. Bioinformatics analyses suggested that mRNAs encoding kinesin heavy chain 73 (KHC73), a protein that belongs to Kinesin-3 family of anterograde motor proteins, may be a functional target of *miR-92a.* Behavioral studies that employed expression of *khc73* with and without its 3’ untranslated region (UTR) containing *miR-92a* target sites, luciferase assays in HEK cells with reporters containing wild-type and mutant target sequences in the *khc73* 3’UTR, and immunohistochemistry experiments involving KHC73 expression with and without the wild-type *khc73* 3’UTR, all point to the conclusion that *khc73* is a major target of *miR-92a* in its functional role as a miRNA memory suppressor gene.

## Significance Statement

Much remains to be learned about how microRNAs (miRNAs) regulate gene expression for cognitive processes such as memory formation. The important questions include which of the many different miRNAs are involved, what are their targets, and what specific aspects of memory formation do they regulate? Here, we show that the miRNA, *miR-92a*, normally functions to suppress the consolidation of memories by repressing the expression of a specific kinesin molecule, kinesin heavy chain 73 (*khc73*), in the mushroom body neurons (MBn) of the *Drosophila* brain.

## Introduction

MicroRNAs (miRNAs) are small non-coding RNAs that regulate gene repression post-transcriptionally. Since their discovery less than two decades ago, miRNAs have been implicated in diverse biological processes and diseases. The *miR-17-92* cluster in humans was one of the earliest miRNAs discovered. It is overexpressed in certain types of cancer and, thus, named *oncomiR-1* (He et al., 2005). In addition to its function as an oncogene, this cluster of miRNAs has been implicated in development, immune disease, neurodegenerative diseases and aging (Mogilyansky and Rigoutsos, 2013). The nervous system functions of *miR-17-92* cluster include the regulation of axonal outgrowth in embryonic cortical neurons (Zhang et al., 2013), hippocampal neurogenesis, and modulation of anxiety, depressive-like behavior, and pain in response to nerve injury in adult rodent models (Jin et al., 2016; Sakai et al., 2017).

Six miRNAs comprise the *miR-17-92* cluster, with the functions of individual miRNAs in the cluster generally remaining unclear. However, one miRNA in the cluster, *miR-92a,* regulates neuronal progenitor cell divisions and differentiation during the development of the mouse embryonic neocortex and division of *Drosophila* neuroblasts (Bian et al., 2013; Yuva-Aydemir et al., 2015), suggesting conservation of function. A role for *miR-92a* in homeostatic synaptic scaling by repressing rat hippocampal GluA1 subunits of AMPA receptors has been reported (Letellier et al., 2014). In addition, the levels of *miR-92a* cycle in adult *Drosophila* pigment-dispersing factor (PDF) neurons across day and night, thus underlying the rhythmicity in excitability of these neurons (Chen and Rosbash, 2017).

Here, we show that *miR-92a* suppresses *Drosophila* olfactory memory consolidation by constraining the expression of *kinesin heavy chain 73* (*khc73*). *khc73* is homologous to the mammalian *Kif13b/GAKIN/*Kinesin-3 family of proteins that have plus end-directed microtubule motor activity. *khc73* is expressed specifically in the nervous system by late stages of *Drosophila* embryogenesis (Li et al., 1997) and has a role in asymmetric neuroblast division (Siegrist and Doe, 2005). Kinesin family members also participate in the transport of cargo in axons (Horiguchi et al., 2006), maturation of presynaptic release sites and synaptic function (Tsurudome et al., 2010).

Since restricting *miR-92a* expression enhances memory consolidation, its normal function must be to suppress consolidation. Hence, *miR-92a* is characterized as a memory suppressor gene, which we define as any gene that enhances memory performance when removed functionally from an organism. We show that the memory suppressor function is specific to a form of consolidated memory named anesthesia resistant memory (ARM) and that this suppressing function maps to the αβ and γ mushroom body neurons (MBn), neurons known to have dominant roles in olfactory memory formation (Heisenberg, 2003; Guven-Ozkan and Davis, 2014). Opposite to *miR-92a,* we identify *khc73* as a memory promoting gene, enhancing memory consolidation when overexpressed. We present other results showing that *khc73* is a target of *miR-92a*. From a broader perspective, our results indicate that memory consolidation requires anterograde motor proteins, presumably by transporting synaptic cargo from the soma to synapses mediating consolidation.

## Materials and Methods

### Fly stocks

Flies were cultured using standard methods. The flies used for *miR-92a* inhibition (*UAS-miR-92aSP*) contained two copies of the sponge transgene, one copy on the second chromosome and another on the third. Similarly, the *UAS-scrambled* control flies carried a double insertion of the transgene on chromosomes 2 and 3 (Loya et al., 2009; Fulga et al., 2015). *miR-92a* genomic deletions were as described (Yuva-Aydemir et al., 2015). Other *UAS* transgenic lines included *UAS-miR-92a* (Yuva-Aydemir et al., 2015), *UAS-khc73, UAS-HA-khc73* (Siegrist and Doe, 2005), *UAS-HA-khc73^+3’UTR^* (Tsurudome et al., 2010), VDRC RNAi lines, and *UAS-dcr-2* (Dietzl et al., 2007). *GAL4* lines used include *1471*, *c155*, *c305a*, *c739*, *Gad*, *GH146*, *MB-GeneSwitch* (*247-GeneSwitch*), *MZ604*, *nSyb*, *R11D09*, *R13F02*, *R25H11*, *R28H05*, *R35B12*, *Repo*, *TH*, and *VT64246*.

### Behavior

One- to 5-d-old flies were used for standard two-odor discriminative aversive conditioning paradigm (Beck et al., 2000; Busto et al., 2015; Guven-Ozkan et al., 2016). The odorants used were benzaldehyde (ben) and 3-octanol (oct). Memory was tested using a T-maze. For ARM experiments, anesthesia was induced at different time points after conditioning by transferring flies to glass vials and keeping them on ice for 2 min followed by recovery in regular food vials. For gene switch experiments, flies were fed on food containing 200 μm RU486 (Mifepristone-Sigma) to regulate *UAS*-driven transgene expression. Memory retention, acquisition, odor, and shock avoidance experiments were performed as described (Busto et al., 2015; Guven-Ozkan et al., 2016).

### Bioinformatics and statistical analyses

Putative mRNA targets for *miR-92a* were predicted using online tools DIANA-microT-CDS (Paraskevopoulou et al., 2013), TargetScan (Lewis et al., 2005; Ruby et al., 2007), and miRecords (Xiao et al., 2009). The pipeline was designed to select the candidate genes with strong prediction by multiple algorithms. Human *miR-92a* target genes with homology to *Drosophila* targets were selected using DIOPT (Hu et al., 2011). GraphPad Prism was used for statistical analyses. Two sample, two-tailed Student’s *t* tests were used to compare two conditions. For multiple group comparisons, one-way ANOVA followed by Dunnett’s or Bonferroni’s *post hoc* tests were used.

### Molecular cloning and mutagenesis

To generate *khc73* luciferase reporter, ∼2.7 kb of 3’ untranslated region (UTR) was PCR amplified from *UAS-khc73^+3’UTR^* genomic DNA and cloned into *psiCheck2* vector downstream of *Renilla* luciferase using the In-Fusion enzyme from Clontech. Three *miR-92a* sites were mutagenized sequentially to generate the triple mutant with an ATAAGCT sequence substituted to GCGGTAC. The triple mutant served as a negative control for *miR-92a* and *miR-310* repression.

### Luciferase assay

HEK293T cells were seeded into 96-well microtiter plates 16 h before transfection. A total of 400 ng of wild-type or triple mutant *psiCheck2-khc73* 3’UTR constructs were transfected using QIAGEN PolyFect Transfection Reagent and a final concentration of 500 nm of the miRNA mimic (Dharmacon). *Drosophila miR-310* served as positive and *Caenorhabditis elegans miR-239b* as negative controls. Luciferase substrate of the Dual-Glo Luciferase Assay System (Promega) was used to activate firefly signal followed by Stop&Glo substrate to inhibit firefly and activate *Renilla* subsequently. A CLARIOstar plate reader from BMG Labtech was used to measure firefly and *Renilla* luminescence. *Renilla* luciferase activity was normalized to firefly. *Renilla*/firefly ratios for *miR-92a* and *miR-310* transfections were normalized to the *miR-239b* negative control for both wild-type and triple mutant reporters, independently.

### Immunohistochemistry

We followed the protocol described earlier except that we incubated tissue with primary antibodies for 2 d (Cervantes-Sandoval et al., 2016; Guven-Ozkan et al., 2016). The primary antibodies used were mouse anti-HA (1:500, 16B12 Thermofisher) and rabbit anti-Scribble (1:500; Cervantes-Sandoval et al., 2016). Secondary antibodies include Alexa Fluor 488 goat anti-mouse (1:500) and Alexa Fluor 633 goat anti-rabbit (1:500). Images were obtained using Leica TCS SP8 confocal microscope. Regions of interest (ROIs) were defined around MB horizontal lobes or antennal lobes. Mean signal intensities of maximum projection images for eighth Z-sections (3 μm each) encompassing the anterior/posterior extent of the MBs were measured using ImageJ. Signal from the antennal lobes was subtracted from MB signal for background normalization. Ratios of HA and Scribble signals were calculated to minimize brain-to-brain variability. Two sample, two-tailed Student’s *t* tests were used to compare *scrambled* to *miR-92aSP* brains.

## Results

### *miR-92a* inhibition enhances aversive olfactory memory

**Figure 1.**
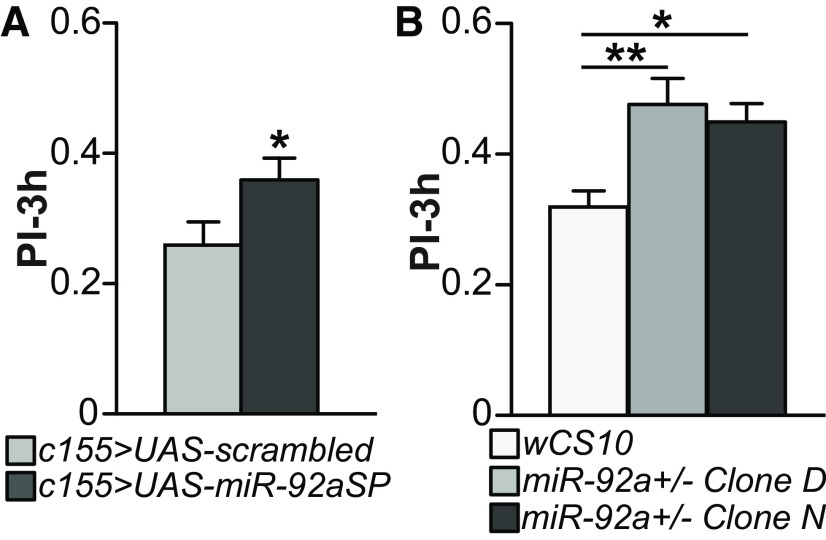
*MiR-92a* inhibition enhances 3-h olfactory memory. ***A***, *MiR-92a* sponge expression enhances 3-h aversive memory. Pan-neuronal inhibition of *miR-92a* by expressing a *UAS-miR-92a sponge* (*UAS-miR-92aSP*) transgene enhances 3-h memory. The *UAS-scrambled* transgene was used as the control. PI: performance index, PIs are the mean ± SEM with *n* = 20. Two-tailed, two-sample Student’s *t* test, *p < 0.05. ***B***, *MiR-92a* heterozygous flies exhibit enhanced memory performance. Two lines of a *miR-92a* deficiency exhibit enhanced PIs compared with a *wCS10* control. PIs are the mean ± SEM with *n* = 10. One-way ANOVA followed by Dunnett’s *post hoc* tests, *p < 0.05, **p < 0.01.

Employing miRNA sponge transgenes offers a versatile approach to inhibit miRNAs in a time and cell specific manner. We previously screened a large collection of *Drosophila* miRNA sponge transgenic lines for aversive olfactory memory using the pan-neuronal driver, *c155-GAL4* (Busto et al., 2015, 2016, 2017; Guven-Ozkan et al., 2016). Our primary screen identified *miR-92a* as a memory suppressor miRNA since we observed elevated memory performance on reducing the expression of *miR-92a* with two independent *UAS-miR-92a sponge* transgenes (*miR-92aSP*). However, further tests failed to consistently reproduce these initial effects (Busto et al., 2015). We reasoned that this variability might be because of weak inhibition, so we tested the effect of expressing two copies of *miR-92aSP* with the *c155-GAL4* driver. The double sponge caused a significant increase in 3-h memory compared with *c155>scrambled* control flies (Fig. 1*A*). To confirm that the memory enhancing effects of expressing *miR-92aSP* are because of reducing *miR-92a* expression, we tested heterozygous adult *miR-92a* knock-out flies to mimic the hypomorphic effects of *miR-92aSP* knock-down (Fig. 1*B*). We backcrossed two *miR-92a* knock-out lines (named D and N; Yuva-Aydemir et al., 2015) into a *wCS10* background and employed *wCS10* flies as control. Both D and N clones of the *miR-92a*+/− heterozygotes showed significantly enhanced memory, similar to that observed with *miR-92a sponge* (*miR-92aSP*) flies. Neither *miR-92aSP* animals nor *miR-92a*+/− heterozygotes exhibited changes in odor and shock avoidance compared with their respective controls (Extended Data Fig. 1-1).

### *miR-92a* functions in MB αβ and γ neurons during development and adulthood

**Figure 2.**
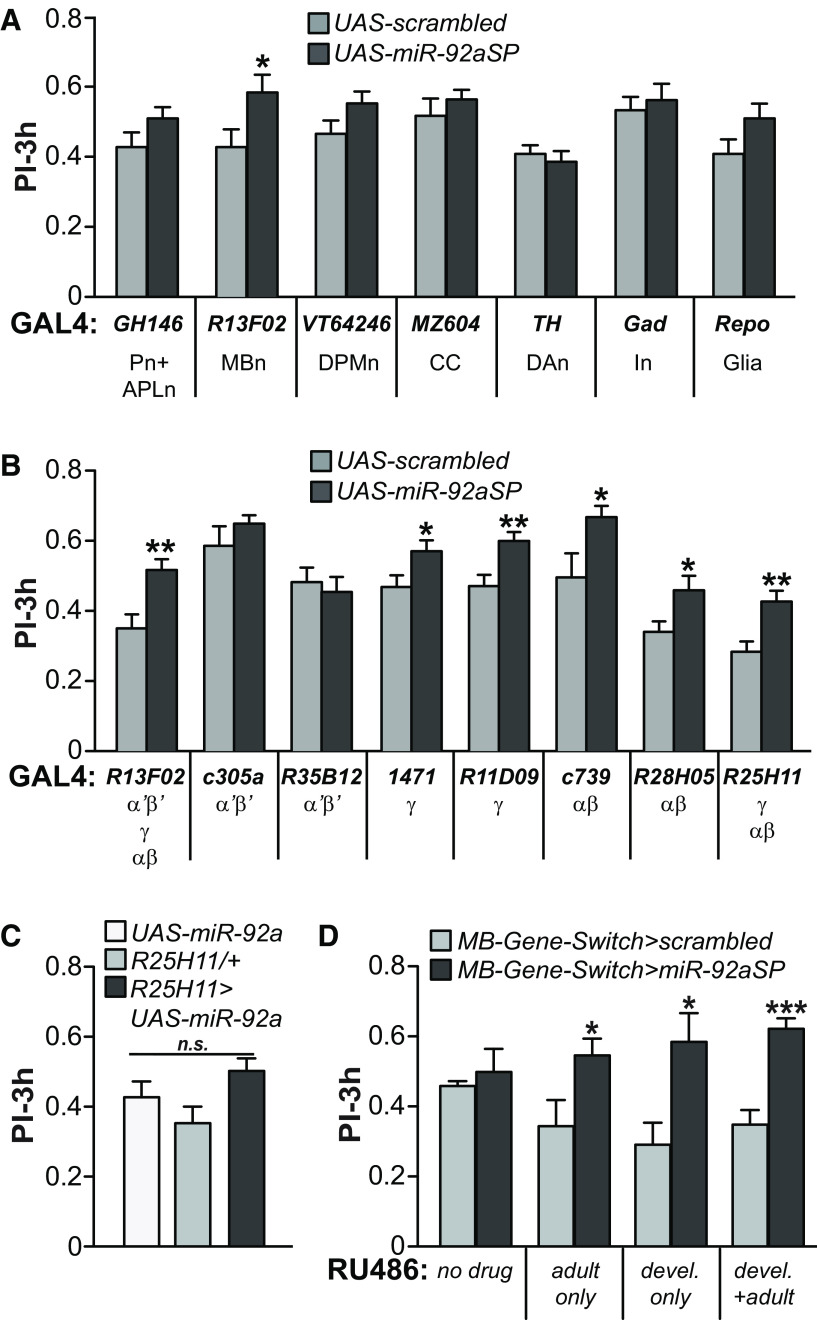
Memory enhancement from inhibiting *miR-92a* in MBns. ***A***, *MiR-92a* inhibition in mushroom bodies enhances 3-h memory. The *UAS-scrambled* and *UAS-miR-92aSP* flies were crossed to a battery of *GAL4* lines that drive expression in specific populations of neurons. Most of the drivers promote expression in neurons of the olfactory pathway. The *GAL4* drivers used and abbreviations of their expression domains are shown below the bar graph. Note that there is some variation in the scores of the control genotypes because of using different *GAL4* drivers. Pn: projection neurons, APLn: anterior paired lateral neuron, DPMn: dorsal paired medial neuron, CC: central complex, DAn: dopaminergic neurons, In: inhibitory neurons. PIs are the mean ± SEM with *n* = 12. Two-tailed, two-sample Student’s *t* tests for each driver expressing *miR-92aSP* compared with expression of the scrambled control, *p < 0.05. ***B***, *MiR-92a* spatial mapping to subsets of MBns. Two independent drivers were used for each type of MBn: α’β’, γ, and αβ. The *GAL4* drivers employed and their expression in the three broad types of MBns are shown below the graph. PIs are the mean ± SEM with *n* = 12. Two-tailed, two-sample Student’s *t* tests for each driver expressing *miR-92aSP* compared with expression of the scrambled control, *p < 0.05, **p < 0.01. ***C***, Overexpression of *miR-92a* does not affect 3-h memory. *MiR-92a* was overexpressed in αβ and γ MBns using *R25H11-GAL4*. Three-hour olfactory memory performance of *R25H11-GAL4*>*UAS-miR-92a* flies was compared with *GAL4-*only and *UAS*-only controls. PIs are the mean ± SEM with *n* = 14. One-way ANOVA followed by Bonferroni’s *post hoc* tests. n.s., not significant. ***D***, Memory enhancement occurs from *miR-92a* inhibition during both development and adulthood as assayed using the Gene-Switch system. Administration of RU486 during development (devel), adulthood (adult), or both (devel + adult) modulates mushroom body expression of *miR-92aSP* controlled by the MB-Gene-Switch driver. Note that RU treatment either during development or adulthood alters the PIs of the control genotype. PIs are the mean ± SEM with *n* = 6–8. Two-tailed, two-sample Student’s *t* tests for each RU486 feeding condition, *p < 0.05, ***p < 0.001.

To delineate the subset of neurons in which *miR-92a* functions as a memory suppressor, we crossed *miR-92aSP* flies with a battery of *GAL4* drivers expressed in different neurons of the olfactory nervous system (Guven-Ozkan and Davis, 2014). We also included a glia specific *GAL4* driver, *Repo-GAL4*. Among all the drivers tested, inhibition of *miR-92a* in MBn using *R13F02-GAL4* resulted in a memory performance that was significantly elevated compared with the respective *scrambled* control (Fig. 2*A*), without altering shock or odor perception (Extended Data Fig. 2-1*A*). MBn can be classified into three main subtypes based on the trajectory of their axons. Axons of MB αβ and α’β’ neurons bifurcate to form both vertical and horizontal branches whereas γ neurons have only a horizontal branch (Crittenden et al., 1998). We used two independent drivers for each MB subtype to inhibit *miR-92a* in a neuron subtype-specific manner (Fig. 2*B*) along with *R13F02-GAL4* for pan-MBn expression. Inhibiting *miR-92a* in MB αβ and γ neurons enhanced memory significantly using *1471-GAL4* and *R11D09-GAL4* for γ neurons and *c739-GAL4* and *R28H05-GAL4* for αβ neurons (Fig. 2*B*) without altering odor or shock avoidance (Extended Data Fig. 2-1*B*,*C*). *R25H11-GAL4*, expressed in both γ and αβ neurons, produced the same memory enhancing effect, further strengthening this observation (Fig. 2*B*; Extended Data Fig. 2-1*D*). In contrast, *miR-92aSP* expression in α’β’ MBn was without effect (Fig. 2*B*). To test the effect of increasing *miR-92a* expression in γ and αβ MBn, we crossed a *UAS-miR-92a* overexpressing transgene with *R25H11-GAL4.* This genotype exhibited memory performance at the same level as *UAS-* and *GAL4-*only controls (Fig. 2*C*), indicating that *miR-92a* is expressed at saturating levels in control flies for memory phenotypes.

10.1523/ENEURO.0224-20.2020.f1-1 Extended Data Figure 1-1 Odor and shock avoidance of *miR-92aSP-*expressing and *miR-92a* heterozygous flies. ***A***, The *c155-GAL4>UAS-miR-92aSP* flies avoided ben, oct and electric shock stimuli indistinguishably from *c155*>*UAS-scrambled* flies. Avoidance Index: AI, AIs are the mean ± SEM with *n* = 6. Two-tailed, two-sample Student’s *t* tests for each condition. No significant differences were detected. ***B***, *miR-92a* heterozygotes for Clones D and N flies avoided ben, oct and electric shock stimuli indistinguishably from the *wCS10* control. AIs are the mean ± SEM with *n* = 6. Two-tailed, two-sample Student’s *t* tests for each condition. No significant differences were detected. Download Figure 1-1, EPS file.

10.1523/ENEURO.0224-20.2020.f2-1 Extended Data Figure 2-1 Odor and shock avoidance of *miR-92aSP* and *khc73* overexpression flies. ***A–D***, Flies carrying *UAS-miR-92aSP* driven by *R13F02-GAL4*, *R11D09-GAL4*, *R28H05-GAL4*, or *R25H11-GAL4* avoided ben, oct, and electric shock stimuli indistinguishably from flies carrying *UAS-scrambled* and the same drivers. AIs are the mean ± SEM with *n* = 6. Two-tailed, two-sample Student’s *t* tests for each condition. No significant differences were detected. ***E***, *R25H11-GAL4>UAS-khc73* animals avoided ben and oct indistinguishably from *GAL4*-only and UAS-only controls. The *UAS-khc73/+* control group exhibited an increased avoidance of shock stimuli compared to the *R25H11/+* control and *R25H11-GAL4>UAS-khc73* experimental flies. This increased avoidance fails to explain the memory enhancement of the experimental genotype. AIs are the mean ± SEM with *n* = 6. One-way ANOVA followed by Bonferroni’s *post hoc* tests, *p < 0.05. Download Figure 2-1, EPS file.

We chose to use the Gene-Switch System to dial *miR-92aSP* expression up or down in MBn at different stages of development (McGuire et al., 2004) to delineate developmental versus adult roles. A MBn-specific Gene-Switch *GAL4* was crossed to *miR-92aSP* flies and the progeny were fed the ligand RU486 at different stages to activate the Gene-Switch *GAL4*. Flies raised on RU486-laced food throughout the lifespan served as a positive control while flies raised on non-supplemented food served as negative control (Fig. 2*D*). Surprisingly, *miR-92a* inhibition during development or only during adulthood enhanced 3-h olfactory memory in adult flies compared with flies expressing *scrambled* sequences. We also tested *miR-92aSP* memory effect using TARGET system attempting to strengthen our conclusion (McGuire et al., 2004). However, the *GAL4* drivers used, in general, failed to reproduce the memory phenotype even when the animals were kept at 30°C during both developmental and adult stages (data not shown). The reason for this failure is unknown, although it is possible that the drivers employed were simply not strong enough to overcome the repressive effects of *GAL80^ts^* at the permissive temperature of 30°C.

### *miR-92a* inhibition enhances intermediate-term memory

**Figure 3.**
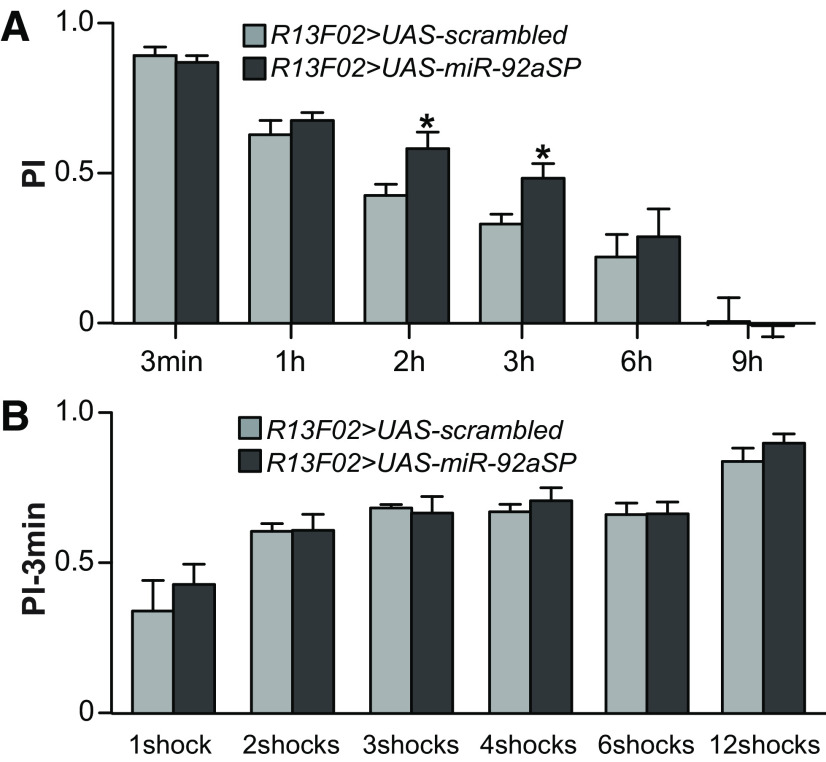
Inhibiting *miR-92a* enhances intermediate-term memory. ***A***, Memory decay in flies expressing *miR-92aSP* in the mushroom bodies. Three-minute, 1-, 2-, 3-, 6-, and 9-h memory was tested in flies expressing *UAS-miR-92aSP* or *UAS-scrambled* under the control of *R13F02-GAL4*. PIs are the mean ± SEM with *n* = 6–8. Two-tailed, two-sample Student’s *t* tests between the groups at each time point, *p < 0.05. ***B***, No effect of *miR-92a* inhibition on memory acquisition. Three-minute memory of *R13F02-GAL4>UAS-scrambled* or *R13F02-GAL4>UAS-miR-92aSP* flies tested after 1, 2, 3, 4, 6 and 12 shock-training paradigms. PIs are the mean ± SEM with *n* = 6. Two-tailed, two-sample Student’s *t* tests for each shock group of flies expressing *miR-92aSP* compared with expression of the scrambled control. No significant differences were found.

We tested the memory of *miR-92aSP* flies at different times after aversive conditioning to dissect the phase of memory enhanced by *miR-92a* inhibition. Memory was not altered at 3-min or 1-h after conditioning using the pan-MBn driver *R13F02-GAL4* but was enhanced, relative to control flies, at 2 and 3-h after conditioning. This enhancement disappeared by 6-h (Fig. 3*A*). We replicated and refined this observation by performing the same time course experiment (Extended Data Fig. 3-1*A*) with drivers that promote expression in the γ (*R11D09*) and the αβ neurons (*R28H05*). These experiments reveal clearly that *miR-92a* inhibition enhances memory at intermediate time points. To further exclude a possible role for *miR-92aSP* in memory immediately after conditioning, hidden by a ceiling effect, we tested 3-min memory performance after training flies with varying numbers of shock pulses to titrate training intensity (Fig. 3*B*; Extended Data Fig. 3-1*B*). Although performance increased with an increasing number of shock pulses, there was no difference in performance scores between the control and experimental genotype using all three *GAL4* drivers (Fig. 3*B*; Extended Data Fig. 3-1*B*). These data lead to the strong conclusion that *miR-92a* inhibition enhances intermediate-term memory without effect on memory acquisition.

10.1523/ENEURO.0224-20.2020.f3-1 Extended Data Figure 3-1 *MiR-92aSP* memory retention, acquisition and cold shock experiments with mushroom body γ and αβ drivers. ***A***, *miR-92a* inhibition enhances intermediate-term memory. Inhibiting *miR-92a* in γ MBns using *R11D09-GAL4* enhances 1- and 3-h memory. Inhibiting *miR-92a* in αβ MBns using the *R28H05-GAL4* driver enhances 1-, 3-, and 6-h memory. The PI for *UAS-miR-92aSP*-expressing flies was compared to the PI for *UAS-scrambled*-expressing flies at each time point. PIs are the mean ± SEM with *n* = 12. Two-tailed, two-sample Student’s *t* tests for each time point, *p < 0.05, **p < 0.01. ***B***, *miR-92a* inhibition does not affect acquisition. Three-minute memory of *R11D09-GAL4* or *R28H05-GAL4* driven *miR-92aSP* flies tested after receiving varying numbers of shock pulses with a 1-min CS+ odor presentation. PIs are the mean ± SEM with *n* = 6–12. Two-tailed, two-sample Student’s *t* tests. No significant differences were detected. ***C***, Inhibiting *miR-92a* in γ and αβ MBns enhances ARM. Flies were subjected to cold shock 2 h after aversive conditioning, and memory was tested at 3 h. Memory of *R11D09-GAL4* and *R28H05-GAL4* driven *miR-92aSP* flies was tested with or without cold shock treatment. PIs are the mean ± SEM with *n* = 16–20. Two-tailed, two-sample Student’s *t* tests, *p < 0.05, **p < 0.01. Download Figure 3-1, EPS file.

### *miR-92a* inhibition enhances memory consolidation

**Figure 4.**
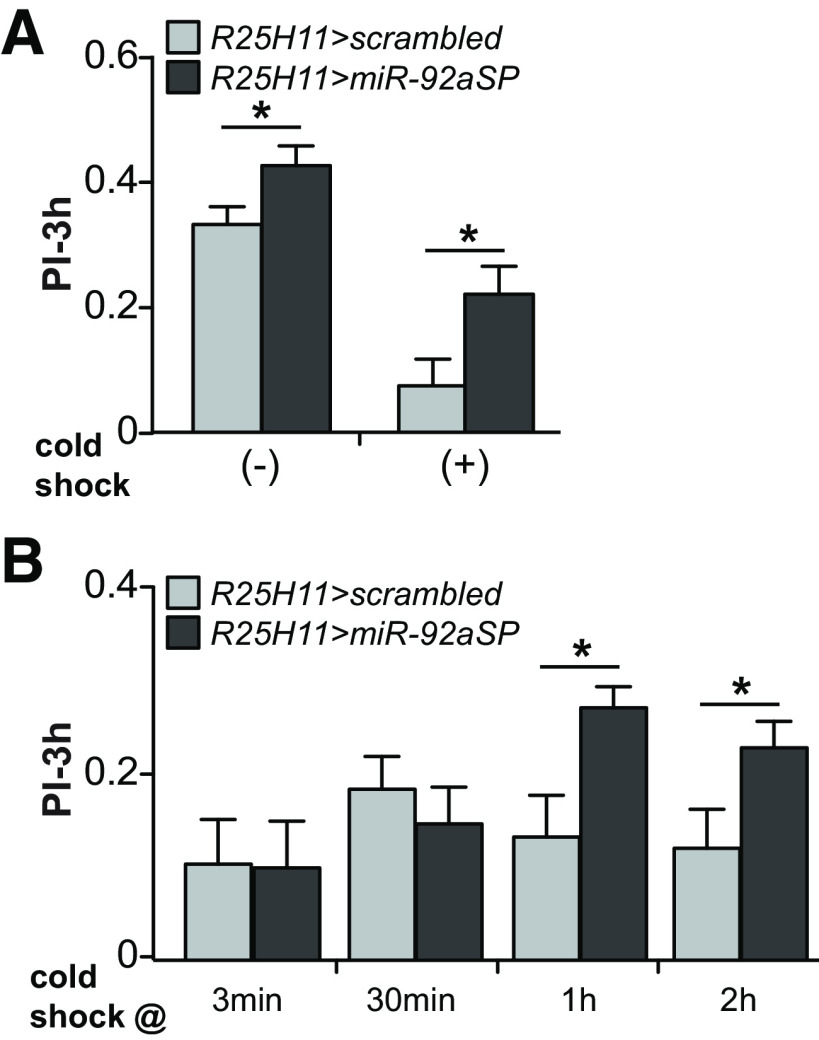
Inhibiting *miR-92a* enhances memory consolidation. ***A***, Three-hour memory performance of *R25H11-GAL4>UAS-scrambled* or *R25H11-GAL4>UAS-miR-92aSP* flies with or without cold shock induced amnesia applied 2-h after aversive training. Cold-shocked flies exhibited increased performance, indicating enhanced ARM. PIs are the mean ± SEM with *n* = 14–16. Two-tailed, two-sample Student’s *t* tests, *p < 0.05. ***B***, Three-hour memory performance of *R25H11-GAL4>UAS-scrambled* or *R25H11-GAL4>UAS-miR-92aSP* flies subjected to cold shock induced amnesia at various time points after aversive training. PIs are the mean ± SEM with *n* = 12–16. Two-tailed, two-sample Student’s *t* tests, *p < 0.05.

To further dissect the intermediate-term memory phenotype, we tested whether *miR-92a* might have a role in memory consolidation. One type of consolidated memory in flies is termed ARM (Quinn and Dudai, 1976; Tempel et al., 1983), traditionally studied by subjecting flies to a 2-min cold shock to trigger amnesia after training and remove anesthesia sensitive memory (ASM). The memory remaining after this insult is then measured at later time points. We measured 3-h memory of *R25H11>miR-92aSP* flies with or without cold shock given at 2-h after training. Interestingly, *miR-92a* inhibition enhanced ARM compared with the *scrambled* control flies indicating a role for *miR-92a* in consolidation of this form of memory (Fig. 4*A*). We reproduced and extended this conclusion by assaying ARM in two other genotypes that express *miR-92aSP* in the γ and the αβ neurons (Extended Data Fig. 3-1*C*). Moreover, we studied the time course of ARM enhancement by applying the cold shock at various times after training with testing occurring at 3-h. No ARM enhancement was observed with cold shock at 3 or 30-min after training, but cold shock at 1 or 2-h provided the maximal enhancement of ARM (Fig. 4*B*). This indicates that *miR-92a* disruption impacts memory consolidation processes that occur between 30–120-min after conditioning.

### *miR-92a* target prediction pipeline identifies *khc73* as a potential mRNA target of *miR-92a*

**Figure 5.**
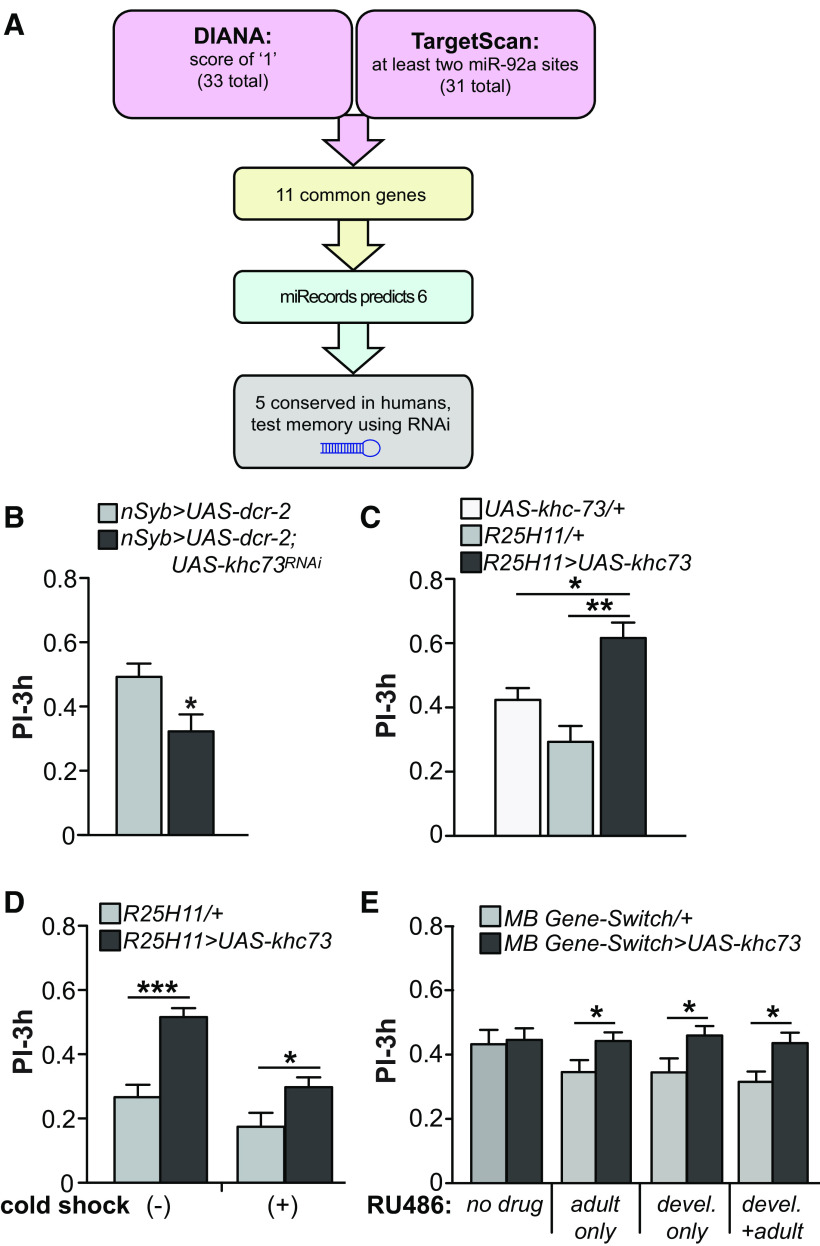
*MiR-92a* mRNA target prediction. ***A***, Design of the mRNA target prediction pipeline. Top predictions for DIANA and TargetScan have 11 genes in common, six of which are also predicted by miRecords. Five of these potential mRNA targets have human homologs that are predicted targets for human *miR-92a*. ***B***, *khc73 RNAi* expression produces memory impairment. Only *khc73* of the five candidates (Table 1) significantly impaired 3-h memory using RNAi driven by pan-neuronal *nSyb-GAL4*. PIs are the mean ± SEM with *n* = 10. Two-tailed, two-sample Student’s *t* tests, *p < 0.05. ***C***, *UAS-khc73* overexpression enhances 3-h memory. Expression of *UAS-khc73* driven by *R25H11-GAL4* significantly enhanced 3-h memory compared with *UAS*-only and *GAL4-*only controls. PIs are the mean ± SEM with *n* = 10. One-way ANOVA followed by Bonferroni’s *post hoc* tests, *p < 0.05, **p < 0.01. ***D***, Overexpressing *khc73* using *R25H11-GAL4* enhances ARM. Flies were subjected to cold shock 2-h after aversive conditioning and memory was tested at 3-h. Consistent with *miR-92a* inhibition, overexpressing *khc73* enhances ARM. PIs are the mean ± SEM with *n* = 12. Two-tailed, two-sample Student’s *t* tests, *p < 0.05, ***p < 0.001. ***E***, Overexpressing *khc73* during development (devel), adulthood (adult), or both (devel + adult) enhances adult memory as assayed using the Gene-Switch system. Administration of RU486 increases mushroom body expression of *khc73* controlled by the MB-Gene-Switch driver. Similar to the effect observed in Figure 2*D*, RU treatment either during development or adulthood alters the PIs of the control genotype. PIs are the mean ± SEM with *n* = 16–20. Two-tailed, two-sample Student’s *t* tests for each RU486 feeding condition, *p < 0.05.

To identify *miR-92a* target mRNAs and their possible roles in memory consolidation, we first used computational prediction software packages including DIANA-microT-CDS, Targetscan, and miRecords (Lewis et al., 2005; Ruby et al., 2007; Xiao et al., 2009; Paraskevopoulou et al., 2013). To extract the strongest predictions and most likely relevant and conserved targets from among the hundreds predicted by each tool, we employed a stringent pipeline that used all three tools (Fig. 5*A*). DIANA-microT-CDS ranks predicted genes based on an miTG score with the highest score being 1.0. Thirty-three genes were predicted by this tool with a miTG score of 1.0. TargetScan ranks its predictions based on the number of miRNA sites and also on the degree of seed sequence complementarity to predicted sites. We selected 31 genes with at least two predicted *miR-92a* binding sites using this tool. Eleven genes were identified in common using these two different algorithms (Fig. 5*A*). We then determined whether these 11 common genes were predicted by miRecords, which reduced the number of genes of interest to six (Fig. 5*A*). We identified human homologs of predicted *Drosophila* target genes using DIOPT (Hu et al., 2011) and checked whether their mRNA sequences possessed a five or six nucleotide match to the human *miR-92a* seed sequence. Finally, the five final genes that showed this possible conservation of *miRNA*:target interaction in humans were tested to see whether they participated in memory formation by *RNAi* knock-down using a pan-neuronal *nSyb-GAL4* driver (Table 1). The top gene identified by this pipeline, identified from impaired memory scores, was kinesin heavy chain gene, *khc73* (Fig. 5*B*). The impaired memory with RNAi knock-down of *khc73* is opposite of the memory phenotype observed on expressing *miR-92aSP*, which is expected, given that reducing the *miRNA* repressor should increase *khc73* expression.

**Table 1:** Putative *miR-92a* target genes

Gene name	Flybase ID	RNAi line	PI for *nSyb-GAL4* screen	Retest score	*p* value
*CG12024*	*CG12024*	GD-20143	0.45 ± 0.02		
*CG8360*	*CG8360*	GD-41643	0.40 ± 0.08		
*cpr50Ca*	CG13338	KK-100317	0.64 ± 0.07	0.56 ± 0.08	0.15
*khc-73*	CG8183	KK-105984	0.38 ± 0.09	0.28 ± 0.07	0.02*
*crebA*	CG7450	KK-110650	0.37 ± 0.06	0.37 ± 0.08	0.09

Candidate mRNA targets for *miR-92a* were selected by applying the pipeline described in Figure 5*A*. Five genes were screened for a role in memory formation using an RNAi approach. The five lines from the Vienna Drosophila RNAi left (https://stockcenter.vdrc.at/control/main) that were tested are listed. RNAi lines were crossed to the *nSyb-GAL4* driver and tested for 3-h memory with *n* = 4. Each individual RNAi line was compared with a daily *nSyb-GAL4>UAS-dcr-2* control in the respective GD or KK background. The average performance index for the *nSyb-GAL4>UAS-dcr-2* control was 0.36 ± 0.1 for the GD control (*n* = 4) and 0.49 ± 0.04 for the KK control. Three lines with a trend for a significant effect on memory were retested with *n* = 6. Only the *khc-73* RNAi line, had a PI significantly lower than the control (*n* = 10). Results shown are the mean ± SEM. Two-tailed, two-sample Student’s *t* tests, *p < 0.05.

The hypothesis that *khc73* is a target of *miR-92a* predicts that overexpression of *khc73* should enhance memory, mimicking the memory performance of flies with reduced *miR-92a* levels. Consistent with the prediction, we found that *UAS-khc73* overexpression in αβ and γ MBn using *R25H11-GAL4* increased memory performance compared with *UAS-*only and *GAL4-*only controls (Fig. 5*C*), with shock-avoidance and odor-avoidance controls failing to explain this enhancement (Extended Data Fig. 2-1*E*). We made two additional observations from behavioral experiments that provide very significant support for this hypothesis. First, the overexpression of *khc73* in αβ and γ MBn enhanced the same form of memory, ARM, as expression of *miR-92aSP* (Fig. 5*D*). Since there are very few known genetic insults that enhance ARM, the probability that this represents independent processes is extremely small. Second, we overexpressed *UAS-khc73* using the MB-Gene-Switch driver and discovered that increasing *khc73* expression during both developmental and adult stages enhances 3-h memory (Fig. 5*E*). The developmental co-mapping of *khc73* overexpression and *miR-92a* inhibition provides compelling behavioral support for the hypothesis that *khc73* is a primary target of *miR-92a* largely responsible for its memory suppressor function.

### The *khc73* 3’UTR and *miR-92a* binding sites are critical for function

**Figure 6.**
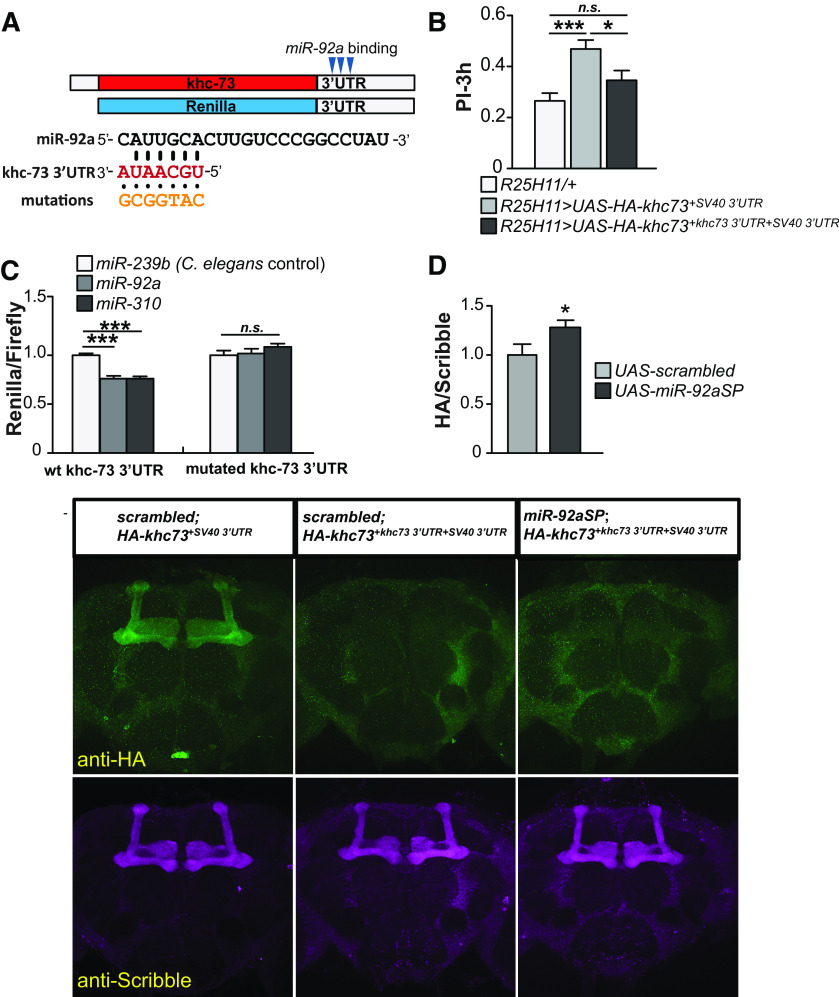
Memory performance of flies overexpressing *khc73* with and without the endogenous 3’UTR. ***A***, Three predicted *miR-92a* binding sites in the endogenous 3’UTR are schematized*. Renilla* luciferase reporter was cloned upstream of the 2.7-kb *khc73* 3’UTR and used for the experiment shown in ***C****.* Nucleotides with predicted *miR-92a* binding are shown in red font. All three *miR-92a* sites were altered to generate a mutant *khc73* 3’UTR that is unresponsive to *miR-92a* repression (shown with orange font) as a negative control for luciferase experiments. ***B***, Overexpressing *khc73* with the endogenous 3’UTR eliminates the memory enhancement gained without the 3’UTR. Expression of HA-tagged *khc73* transgenes with or without 2.9 kb of its own 3’UTR confers normal and enhanced memory performance, respectively. PIs are the mean ± SEM with *n* = 14. One-way ANOVA followed by Bonferroni’s *post hoc* tests, *p < 0.05, ***p < 0.001. ***C***, Overexpressing *miR-92a* represses expression of a *khc73* 3’UTR luciferase reporter. The *Renilla* luciferase signal was normalized to firefly luciferase for each transfection. *C. elegans miR-239b* served as negative and *Drosophila miR-310* as positive controls. *Renilla*/firefly ratios were normalized to negative controls for each reporter construct. Relative repression ratios ± SEM with *n* = 9. One-way ANOVA followed by Bonferroni’s *post hoc* tests, ***p < 0.001, n.s., not significant. wt: wild-type. ***D***, *miR-92a* inhibition derepresses HA-KHC73 signal. *HA-Khc73* transgenes with or without the endogenous 2.9-kb 3’UTR element were overexpressed in MBn using the *R13F02-GAL4* driver. The presence of the endogenous 3’UTR constraints the HA-KHC73 signal. Very weak signals in the mushroom bodies were detected (middle column) and quantified (bar plot). This weak signal was significantly derepressed when the *miR-92aSP* was co-expressed (right column). The bar plot shows the quantification of *R13F02*>*scrambled*, *HA-khc73 + 3’UTR* versus *R13F02>miR-92aSP, HA-khc73 + 3’UTR* brains. ROI was taken from the horizontal lobes by reference to anti-Scribble signal. HA/Scribble ratios were used to minimalize brain to brain variability. Ratios are the mean ± SEM with *n* = 26–30. Two-tailed, two-sample Student’s *t* tests, *p < 0.05.

MiRNAs regulate gene expression post-transcriptionally by binding to sequences in the target gene mRNAs. These binding sites are most often located in the 3’UTR of the mRNAs (Miura et al., 2014). Target site identification software predicts three potential *miR-92a* binding sites in the *khc73* 3’UTR (Fig. 6*A*). We first tested the relevance of the *khc73* 3’UTR to behavior by comparing memory performance of flies that overexpress *khc73* from previously characterized transgenes that include the normal 2.9 kb *khc73* 3’UTR and those that include only the 3’UTR from SV40 with its poly A sequences (Siegrist and Doe, 2005; Tsurudome et al., 2010). Strikingly, memory performance was significantly enhanced when *khc73* was overexpressed with the SV40 3’UTR, but not when overexpressed with the endogenous 2.9-kb 3’UTR (Fig. 6*B*). These results support the idea that the *khc73* 3’UTR provides repressive activity to the memory phenotype and is consistent with the hypothesis that *khc73* expression is repressed by *miR-92a*. To determine whether *khc73* is a direct target for *miR-92a in vitro*, we generated a 3’UTR dual luciferase reporter with *khc73* 3’UTR using the *psiCheck2* vector. The wild-type *khc73* 3’UTR was cloned downstream of *Renilla* luciferase so that expression levels may be controlled by regulatory elements present in the *khc73* 3’UTR (Fig. 6*A*). The *psiCheck2* vector also contains a constitutively expressed firefly luciferase to normalize for transfection efficiency. When the *khc73* 3’UTR-reporter vector was co-transfected with *miR-92a RNA mimic*, a chemically synthesized miRNA, into HEK293T cells, the *Renilla*/firefly luminescence ratio was significantly reduced compared with a *control mimic* (Fig. 6*C*). Previous studies reported that *khc73* 3’UTR is repressed at the *Drosophila* NMJ by the *miR-310-313* cluster of miRNAs which belong to the same family of miRNAs as *miR-92a* (Tsurudome et al., 2010). We thus used one member of the *miR-310* cluster as positive control and found that co-transfecting *miR-310 mimic* resulted in a similar level of repression as *miR-92a* using the *khc73* 3’UTR reporter. To further expand these results and map the repression effects to the *miR-92a* target sequences in the *khc73* 3’UTR, we generated a mutant luciferase reporter construct, mutating the seed sequences of all three *miR-92a/miR-310* sites in the 3’UTR (Fig. 6*A*). The repressive activity observed with *miR-92a* and *miR-310* mimics was not observed using the mutant *khc73* 3’UTR construct, strongly indicating that *miR-92a* repression occurs through the *miR-92a* target sites (Fig. 6*C*). We conclude from our luciferase reporter experiments using cultured cells that *miR-92a* targets sequences in the wild-type *khc73* 3’UTR, providing direct control over the expression of *khc73*.

We obtained a third line of evidence supporting this conclusion by analyzing KHC73 expression *in vivo*. We employed HA-tagged, *khc73* transgenes with or without the 2.9-kb 3’UTR along with *miR-92aSP* and quantified expression by immunohistochemistry (Fig. 6*D*). The *UAS-HA-khc73* transgene carrying only an SV40 3’UTR exhibited robust expression in MBn using *R13F02-GAL4* (Fig. 6*D*, left panel). Importantly, the expression was dramatically reduced when the transgene carried the *khc73* 3’UTR (Fig. 6*D*, middle panel). This observation is consistent with the behavioral data presented above (Fig. 6*B*). When we inhibited *miR-92a* using *miR-92aSP* transgene, we observed a low but significant level of expression (Fig. 6*D*), supporting the conclusion that *miR-92a* represses *khc73* through its 3’UTR *in vivo*. Thus, the behavioral, luciferase reporter experiments and *in vivo* immunohistochemistry all support the conclusion that the *khc-73* 3’UTR provides a target for *miR-92a* repression.

## Discussion

MiRNAs provide impressive regulatory power over gene expression. Individual miRNAs can repress hundreds of mRNAs simultaneously to fine tune the expression of collections of genes post-transcriptionally to regulate biological processes from early development to adult physiology. They function as gene expression rheostats that quickly respond to changing environmental conditions or developmental programs (Ferrante and Conti, 2017). In addition, the expression of miRNAs is altered under circumstances such as stress or disease states. Indeed, miRNAs are widely used as biomarkers for certain diseases and psychiatric conditions and help health care providers in diagnosis of illnesses and in judging efficacy of treatment regimens (Ridolfi and Abdel-Haq, 2017; Lopez et al., 2018). As previously noted, one of the earliest cancer biomarkers identified was the human *miR-17-92* cluster of miRNAs (He et al., 2005), for which *miR-92a* is a member. *miR-92a* has known roles in nervous system development (Bian et al., 2013; Yuva-Aydemir et al., 2015), adult homeostatic synaptic scaling (Letellier et al., 2014), and neuronal excitability (Chen and Rosbash, 2017). Recently, *miR-92a* has been identified as a potential plasma biomarker for Alzheimer’s patients along with two other miRNAs (Siedlecki-Wullich et al., 2019), making *miR-92a* as a notable molecule for further study in memory formation, cognition and disease.

Our data identify a new function of this conserved miRNA in *Drosophila* memory consolidation, extending our knowledge of nervous system functions for *miR-92a* several ways. (1) *miR-92a* is a memory suppressor gene, functioning in this capacity specifically in the αβ and γ MBn. (2) *miR-92a* suppresses a specific process in memory formation, the consolidation of early and labile memory into ARM. (3) The memory suppression effect of *miR-92a* appears to occur from effects during development and during adulthood. One caveat of this provisional conclusion is that the lipophilic nature of the Gene-Switch inducer, RU486, may lead to its storage in fat tissues and subsequent release in adult animals to maintain *miR-92aSP* expression (Mao et al., 2004). (4) The memory suppressor functions of *miR-92a* are mediated largely by the inhibition of the anterograde motor protein, KHC73. (5) *khc73,* itself, functions in the same αβ and γ MBn as *miR-92a* and apparently during both development and adulthood but with opposite effects: promoting consolidation rather than suppressing it.

### *miR-92a* function is very distinct from another suppressor, *miR-980*

*MiR-92a* is not the first identified memory suppressor miRNA. We previously reported that *miR-980* functions as a miRNA memory suppressor gene (Busto et al., 2015; Guven-Ozkan et al., 2016). Our findings reveal a diversity of ways by which memory suppressing miRNAs can function. *miR-980* suppresses memory broadly from functions in many different cell types in the olfactory nervous system, rather than the two types of MBn identified for *miR-92a*. This can be explained by the more general function of *miR-980*: it works by inhibiting the excitable state of neurons, setting a higher threshold for excitation and memory acquisition, rather than suppressing memory consolidation by inhibiting kinesin mediated transport functions as suggested by our current data for *miR-92a*. Moreover, the functions of *miR-980* occur during adulthood, rather than having effects during both development and adulthood as suggested by the Gene-Switch experiments with *miR-92a*. This latter observation is peculiar and poorly understood. It is possible that *miR-92a* participates in developmental processes from its developmental expression and physiological processes from adult expression. If so, the mRNA targets for *miR-92a* during development and adulthood could be similar or completely distinct, although the final developmental and adult effects on olfactory memory map to the same αβ and γ MBn.

### KHC73 may be the primary target for consolidation effects

Given the large number of mRNA targets predicted for any given miRNA and the pleasing concept that miRNAs function to control the expression of groups of genes, it is surprising that our data reveal that the *miR-92a* behavioral effects can largely be explained by the actions of a single target, *khc73*. However, it may be that different physiological or developmental events regulated by miRNAs are in some cases effected by large groups of genes and others by single or small set of gene targets. In addition, for miRNAs implicated in diverse physiological events, it seems likely that the mRNA targets will be distinct according to the event and the cell types that are involved. Moreover, although the *khc73* overexpression memory phenotypes we observed can explain the *miR-92a* inhibition phenotypes, we cannot exclude the possibility that there may be additional genes regulated by *miR-92a* that function in memory formation. Furthermore, the *khc73* 3’UTR harbors predicted sites for other miRNAs. Tsurudome et al. (2010) demonstrated that *miR-310* controls *khc73* expression at NMJ, distinct from the *miR-92a* effects in MBn. Thus, this molecular motor protein is regulated by multiple miRNAs in different cell types.

### Model for *miR-92a* and *khc73* function in MBn for suppression of consolidation

Our data indicate that *miR-92a* suppresses memory consolidation by restricting the expression of *khc73* in the αβ and γ MBn. Given the role of Kinesin-3 type motor proteins in transport, along with prior studies showing that increased KHC73 expression elevates synaptic transmission (Tsurudome et al., 2010), we propose that upregulated KHC73 levels in *miR-92a* inhibited flies increases axonal transport and subsequent neurite function and synaptic transmission. Support for this model is found from studies in other systems. Puthanveettil et al. (2008) showed that stimuli leading to consolidated long-term facilitation of the sensory:motor neuron synapse of Aplysia require increased expression of kinesin heavy chain in both presynaptic and postsynaptic neurons, and that upregulation of kinesin heavy chain in presynaptic neurons is sufficient for the induction of long-term facilitation. These observations, and ours, support the concept that long-term, or more generally “consolidated plasticity,” requires increased kinesin-mediated transport. Our immunohistochemistry experiments reveal that the level of KHC73 when overexpressed with its cognate 3’UTR is surprisingly low compared with when a substitute 3’UTR is used. This suggests that *khc73* expression is under tight post-transcriptional control through miRNA regulation. Interestingly, dimerization facilitates fast, processive movement of KHC73 along microtubules (Huckaba et al., 2011). Therefore, the level of KHC73, as regulated by *miR-92a*, may be the rate-limiting step for fast motor complexes to form. Increased KHC73 levels, because of environmental factors, developmental programs, or other elements that influence *miR-92a* function would release the brake on cargo transport leading to higher neuronal function and performance.

**Figure 7.**
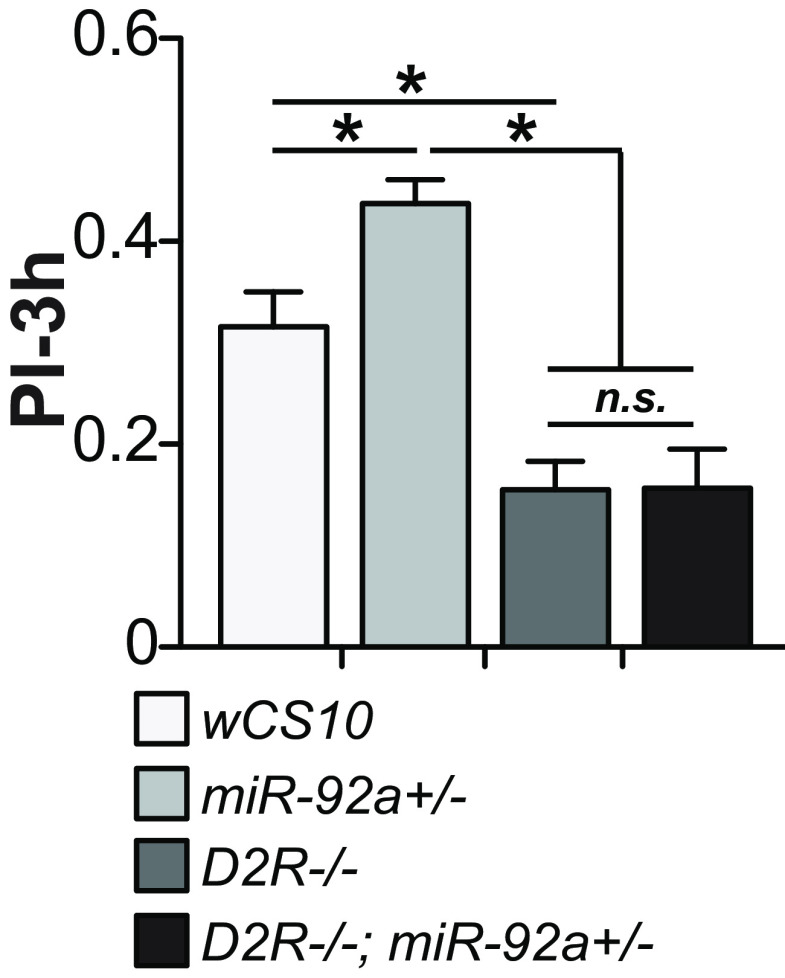
Memory enhancement of *miR-92a*+/− mutant flies requires *D2R*, type 2 dopamine receptor. Three-hour memory scores of *miR-92a*+/−, *D2R*−/−, and *D2R*−/−*; miR-92a*+/− double mutants were tested and compared with *wCS10* control using one-way ANOVA followed by Bonferroni’s *post hoc* tests, *p < 0.05, n.s., not significant. PIs are the mean ± SEM with *n* = 20–23.

Our analysis pipeline for identifying *miR-92a* mRNA targets, in which we used human conservation as a criterion, identified five genes. There are *miR-92a* seed matches in human *Kif13b*, which is homologous to *Drosophila khc73*, two with identical six base pair matches in the coding sequence and one with a 5/6 base pair match in the 3’UTR. It would be interesting to test whether mammalian *miR-92a* might be involved in memory processes by regulating *Kif13b* or kinesin genes highly related to *Kif13b*. Another broad issue for future investigation surrounds the identity of other molecules that may function with *miR-92a* and *khc73* in ARM consolidation. Interestingly, the phenotypes we observed for *miR-92a* knock-down have an intriguing relationship to those for knock-out of the type 2 dopamine receptor, *D2R.* The *D2R* receptor and *miR-92a* are both involved in ARM consolidation in the same MB subtypes (Scholz-Kornehl and Schwärzel, 2016), with *D2R* knock-out impairing memory while *miR-92a* inhibition enhances memory. We tested for possible genetic interactions by epistasis experiments. The memory enhancement of *miR-92a*+/− mutants was abolished and indistinguishable from *D2R*−/− mutants when we tested the double mutants (Fig. 7) suggesting a possible genetic interaction. It would be interesting to explore the mechanistic details of how *miR-92a-*mediated memory enhancement is dependent on dopamine signaling in the future.

## Synthesis

Reviewing Editor: Michael Michaelides, NIDA-NIH Decisions are customarily a result of the Reviewing Editor and the peer reviewers coming together and discussing their recommendations until a consensus is reached. When revisions are invited, a fact-based synthesis statement explaining their decision and outlining what is needed to prepare a revision will be listed below. The following reviewer(s) agreed to reveal their identity: André Fiala.

Reviewer 1

The authors report that impairing/mutating a specific miRNA enhances memory consolidation in Drosophila. In particular, the behavioral expression of anesthesia-resistant memory is enhanced. As a potential target kinesin heavy chain 73 was identified that appears to be supressed by miRNA-92a. The molecular mechanisms underlying distinct forms of longer-lasting memories are only superficially understood. Therefore, the data reported here represent an important and novel piece of information.

I have read the study very carefully. All experiments appear well done, and conclusions also are logical. I have tried my best to find any errors, but actually all experiments are conducted according to the standards in the field. The sample sizes are okay, the statistical tests are sound, all controls are at place, and the conclusions are drawn with care and in a cautious way. I recommend that this interesting manuscript will be published as it is.

Reviewer 2

This is a carefully done study and I have no serious criticism regarding methodology and results. The authors found that reducing expression of miR-92a results in memory enhancement. They also discovered that miR-92a suppresses Khc73 expression by interacting with the khc73 mRNA 3’UTR and inhibiting miR-92a translation. Kinesin heavy chain 73 (Khc73) were shown to play a critical role in consolidation of memory, particularly a memory component called anesthesia resistant memory (ARM). This result is consistent with the previous finding in Aplysia that upregulation of kinesin heavy chain in presynaptic neurons is sufficient for the induction of long-term facilitation. It indicates that memory consolidation requires anterograde motor proteins, presumably by transporting synaptic cargo from the soma to synapses.

Based on these findings, the authors claim that miR-92a has the memory suppressor function and call miR-92a a memory suppressor gene. Although the conclusion that miR-92a is a negative regulator of khc73 expression is solid, the authors need to be careful about calling miR-92a a memory suppressor gene, which implies that miR-92a has the memory suppressor function. Since restricting miR-92a expression enhances memory consolidation, some may say that it’s appropriate to call miR-92a has the memory suppressor function and is considered a memory suppressor gene. However, it does not sound right to me. I believe that “memory genes” need to directly control or closely involved in key physiological processes for memory acquisition, consolidation, storage, and retrieval, etc. Does miR-92a actually contribute to memory consolidation by playing a role in dynamic regulation of Khc73 expression under physiological conditions? mir92a is not merely one of the regulatory components of Khc73 expression? I would like the authors to further discuss if and how miR-92a could actually control Khc73 expression in the context of memory processing.

One of the major findings in this study is that Khc73 plays a critical role in consolidation of memory, particularly a memory component called anesthesia resistant memory (ARM). Could this information (involvement of Khc73 in memory consolidation) be included in the manuscript title? For example, “miR-92a suppresses kinesin-mediated memory consolidation in the Drosophila mushroom body neurons”.

A few minor revisions are shown below.

Line 7: “encoding kinesin heavy chain 73 (khc73), a protein that ....”. Proteins are designated by the same symbol as the gene, but in uppercase letters.

Line 330: “When we inhibited miR-92a using miR-92aSP transgene, we observed a low but significant level of expression (Figure 6D), supporting the conclusion that miR-92a represses khc73 through its 3’UTR in vivo”. In Fig. 6D right panel, the image does not clearly show increased HA-khc73 expression in MB with miR-92aSP.

Line 192: more appropriate GeneSwitch references are:

• Osterwalder et al. (2001) A conditional tissue-specific transgene expression system using inducible GAL4. PNAS 98:12596-12601

• Roman et al. (2001) P{Switch}, a system for spatial and temporal control of gene expression in Drosophila melanogaster. PNAS (2001) 98: 12602-1260.

Line 437: “The D2R receptor and miR-92a are both required for ARM consolidation in the same MB subtypes”. Is miR-92a required for ARM consolidation?
